# The Impact of Rapid Weight Loss on Oxidative Stress Markers and the Expression of the Metabolic Syndrome in Obese Individuals

**DOI:** 10.1155/2013/729515

**Published:** 2013-12-19

**Authors:** Eva Tumova, Wensheng Sun, Peter H. Jones, Michal Vrablik, Christie M. Ballantyne, Ron C. Hoogeveen

**Affiliations:** ^1^Centre for Preventive Cardiology, 3rd Department of Internal Medicine, General Teaching Hospital and Charles University in Prague, U Nemocnice 2, 128 08 Prague 2, Czech Republic; ^2^Center for Cardiovascular Disease Prevention, Methodist DeBakey Heart Center, and Section of Cardiovascular Research, Department of Medicine, Baylor College of Medicine, Houston, TX 77030, USA

## Abstract

*Objective*. Obesity is linked with a state of increased oxidative stress, which plays an important role in the etiology of atherosclerosis and type 2 diabetes mellitus. The aim of our study was to evaluate the effect of rapid weight loss on oxidative stress markers in obese individuals with metabolic syndrome (MetS). *Design and Methods*. We measured oxidative stress markers in 40 obese subjects with metabolic syndrome (MetS+), 40 obese subjects without metabolic syndrome (MetS−), and 20 lean controls (LC) at baseline and after three months of very low caloric diet. *Results*. Oxidized low density lipoprotein (ox-LDL) levels decreased by 12% in MetS+ subjects, associated with a reduction in total cholesterol (TC), even after adjustment for age and sex. Lipoprotein associated phospholipase A_2_ (Lp-PLA_2_) activity decreased by 4.7% in MetS+ subjects, associated with a drop in LDL-cholesterol (LDL-C), TC, and insulin levels. Multivariate logistic regression analysis showed that a model including ox-LDL, LpPLA_2_ activity, and myeloperoxidase (MPO) improved prediction of MetS status among obese individuals compared to each oxidative stress marker alone. *Conclusions*. Oxidative stress markers were predictive of MetS in obese subjects, suggesting a higher oxidative stress. Rapid weight loss resulted in a decline in oxidative stress markers, especially in MetS+ patients.

## 1. Introduction

Obesity is one of the leading causes of overall morbidity and mortality in Western societies and the prevalence of obesity continues to increase worldwide [1]. It is quickly approaching pandemic proportions, currently afflicting nearly 100 million Americans, with deleterious public health consequences [2]. Recent studies estimated that in 2005 the global population contained 937 million (922–951 million) overweight and 396 million (388–405 million) obese adults, respectively. By 2030, the respective number of overweight and obese adults is projected to be at least 1.35 billion and 573 million individuals [3].

Metabolic syndrome (MetS) is a clustering of abnormalities that includes obesity, arterial hypertension, dyslipidemia, and insulin resistance [4]. Since the original publication of the National Cholesterol Education Program (NCEP) Adult Treatment Panel III (ATP III) diagnostic criteria for MetS [5], two independent studies indicated that more than 1 in 5 adults in the US population meet these diagnostic criteria [6, 7]. Prevalence of MetS increases dramatically from 5%–6% in normal-weight (body mass index (BMI) < 25 kg/m^2^) men and women to 22%–28% in overweight (BMI > 25 kg/m^2^) adults and 50%–60% in obese (BMI ≥ 30 kg/m^2^) individuals [8]. MetS is associated with an increased risk for developing cardiovascular disease and type 2 diabetes mellitus (T2DM) [9]. A meta-analysis of 21 studies [10] demonstrated that MetS is associated with an increase in the risk of death from coronary heart disease (CHD) (relative risk (RR) 1.4; 95% confidence interval (CI) 1.2 to 1.6) and overall mortality (RR 1.7; 95% CI 1.3 to 2.4). Augmented oxidative stress appears to be an important component of both MetS and atherosclerosis and may be an important pathway linking MetS to the increased incidence of CHD and T2DM [11, 12]. Oxidative stress markers are elevated in subjects with established CHD and are associated with obesity and MetS. It was reported that oxidative stress was augmented in otherwise healthy obese subjects with MetS compared with BMI-matched obese individuals without MetS [13].

Oxidative stress is generally considered to be a balance between the production of reactive oxidant species (ROS) and antioxidant capacity. It has been previously reported that antioxidants such as vitamin E, beta-carotene, lycopene, and polyphenolic flavonoids can be associated with various lipoproteins, including low-density lipoprotein (LDL), in the circulation [14]. The impact of these circulating antioxidants on oxidative stress is a topic of ongoing research.

Circulating oxidize LDL (ox-LDL) is generally believed to be proatherogenic, in part because of its lower affinity for the LDL receptor; instead, it is recognized by a variety of scavenger receptors. Uptake of ox-LDL by these scavenger receptors leads to accumulation of ox-LDL within the foam cells of atherosclerotic lesions, where ox-LDL induces endothelial activation and smooth muscle proliferation [15–20]. Plasma ox-LDL levels were found to be significantly higher in obese individuals with MetS [13, 21]. In the Atherosclerosis Risk In Communities (ARIC) Study, circulating ox-LDL levels were adversely associated with individual MetS components and increased risk of T2DM [22]. The Atherosclerosis and Insulin Resistance (AIR) Study reported that ox-LDL levels were associated with both subclinical atherosclerosis, as assessed by ultrasonography, and inflammatory markers, including C-reactive protein (CRP) and tumor necrosis factor-*α* TNF-*α* [23]. Oxidative modification of LDL in the arterial wall is a complex process involving a number of biological pathways. Oxidation can occur through many mechanisms including NADPH oxidases, myoglobin, ROS from the mitochondrial electron transport chain, hemoglobin, oxygenases, and peroxidases, among others [24].

Lipoprotein-associated phospholipase A_2_ (Lp-PLA_2_) catalyzes hydrolysis of the sn-2 ester bond of phospholipids in cellular membranes and lipoproteins (e.g., LDL) in settings of high oxidative stress [25, 26]. Particularly in ox-LDL, in which the phospholipids have undergone extensive oxidative modification, the action of Lp-PLA_2_ results in the release of oxidized nonesterified fatty acids (FA) and lysophosphatidylcholine (lyso-PtdCho) [27]. Lyso-PtdCho and oxidized nonesterified short-chain FA are highly proinflammatory lipid mediators which can stimulate macrophage proliferation, increase the expression of vascular adhesion molecules, and upregulate cytokines. Lp-PLA_2_ is produced by macrophages, and its production is regulated by inflammatory cytokines. In the circulation, 80% of the enzyme is bound to LDL particles, 10–15% to high-density lipoprotein (HDL), and the remaining amount to very low density lipoprotein (VLDL) [28]. Small dense LDL particles (sdLDL) are enriched in Lp-PLA_2_ compared to large buoyant LDL, and Lp-PLA_2_ activity is increased in sdLDL [29, 30]. Lp-PLA_2_ was recently characterized as a novel inflammatory biomarker correlated with several components constituting MetS and implicated in atherosclerosis and incident cardiovascular disease [31, 32].

Myeloperoxidase (MPO) is an enzyme generally believed to be linked to oxidative stress. It is released by leukocytes in a state of inflammation and catalyzes the formation of ROS which play an important role in host defense against microorganisms [33]. On the other hand, various ROS produced by MPO are unstable and promote oxidative modifications of LDL [34]. In particular MPO-derived HOCl modifies apolipoprotein B (apoB), which is accompanied by oxidation of the lipid moiety of LDL and results in the formation of chloramines. The LDL-associated chloramines alter the charge characteristics of LDL particles, leading to the uncontrolled uptake of the modified LDL by macrophages [35]. According to previous studies, antioxidant protection by vitamin E, carotenoids, and lycopene is susceptible to HOCl-mediated LDL oxidation only at relatively high concentrations of HOCl [35]. Interestingly, in vitro supplementation of vitamin E does not protect LDL against HOCl-mediated modification; it may even enhance the oxidation [36]. Vitamin E does not provide effective antioxidant protection against MPO-mediated oxidation of LDL [37]. The only antioxidant able to defend against MPO-mediated LDL oxidation appears to be vitamin C, which scavenges HOCl [38] and regenerates amines from HOCl-derived chloramines [35]. Vitamin C also protects LDL against lipid peroxidation initiated by MPO-derived tyrosyl radicals [39].

Apolipoprotein A-I present on HDL is a selective target for MPO-catalyzed oxidation, converting the lipoprotein into a dysfunctional form [40]. Meuwese et al., in the European Prospective Investigation into Cancer and Nutrition (EPIC-Norfolk) prospective population study, found a positive association of MPO levels with the risk of future CHD in healthy individuals [41].

We have previously shown that moderate acute weight loss of 5–7% with a very low calorie diet (VLCD) in obese subjects with MetS was associated with dramatic improvement in all the clinical components of MetS, even though the individuals remained markedly obese [42]. As a part of this ongoing study, we focus on oxidative stress, as assessed by the measurement of circulating oxidative stress markers, and investigate the relationship between MetS and oxidative stress in obese patients enrolled in a rapid weight loss program.

## 2. Methods

### 2.1. Study Population

Obese individuals (56 women and 24 men, aged 47.1 ± 0.9 years, BMI 38.3 ± 0.7 kg/m^2^) enrolled in a medically supervised rapid weight loss program and 20 lean controls (LC) were recruited for this study during a 12-month period (September 2001–September 2002). The study protocol was approved by the Baylor College of Medicine Institutional Review Board and written informed consent was obtained from each individual. The subjects were classified as MetS positive (MetS+) if they met 3 or more of the NCEP ATP III criteria: waist circumference > 40 in (102 cm) for men or >35 in (88 cm) (original criteria in inches, here listed with centimeter equivalents) for women, triglycerides (TG) ≥ 1.69 mmol/L (150 mg/dL), HDL-C < 1.03 mmol/L (40 mg/dL) for men or < 1.29 mmol/L (50 mg/dL) for women, blood pressure ≥ 130/85 mm Hg or treated hypertension, and fasting glucose ≥ 6.1 mmol/L (110 mg/dL) [7]. However, for subsequent analysis in the current study, we adapted the diagnostic criteria for the MetS revised in 2005, which redefines the fasting glucose criterion as ≥ 5.6 mmol/L (100 mg/dL) [43] (see Supplemental Table 1 in Supplementary Material available online at http://dx.doi.org/10.1155/2013/729515). Sequentially enrolled MetS+ subjects (*n* = 40) were compared with 40 obese subjects matched for age and BMI without MetS (MetS−). The study participants did not receive any medication known to affect glucose tolerance, insulin secretion, or insulin sensitivity during the active weight loss period. Entry criteria for the program included age > 18 years and BMI > 30 kg/m^2^. Exclusion criteria were known eating disorder, cancer, use of lithium or corticosteroids, type 1 diabetes, active inflammatory bowel disease, active gout, liver disease, cardiovascular event within the past 3 months, endocrine causes of obesity, and pregnancy. Diuretic medications were discontinued before entering the program.

The weight loss protocol description is as follows: the weight loss program was offered as a weekly follow-up commitment in an outpatient setting at a tertiary-care medical center site (The Methodist Hospital, Houston, TX, USA) to individuals either self-referred or referred by health-care professionals. Weight reduction was induced by a protein-sparing VLCD of approximately 800 kcal daily consisting of liquid beverages (Nutrimed-Plus, Robard Corp., Mount Laurel, NJ, USA; each serving contains 200 kcal, 6 g of fat, 26 g of protein, and 10 g of carbohydrate) alone or in combination with lean beef, fish, or poultry and daily fluid intake at a minimum of 2 L. Study participants were encouraged not to use any over-the-counter dietary supplements while on the VLCD since the VLCD provided 140% of daily value (%DV) for all essential vitamins (including antioxidant vitamins), minerals, and trace elements.

Antioxidant capacity was not measured in study participants prior to or during the study. The MetS+ individuals had scheduled follow-up visits at 4–6 weeks (visit 2) and 12–20 weeks (visit 3) after the initial baseline evaluation. Six subjects (3 men and 3 women) did not return for their final follow-up visit at visit 3. We used a multiple imputation method [44], a combination of mean and regression imputation, to fill in data for the missing variables on the final visit or these 6 individuals. None of the subjects was on any calorie-restricting diet at baseline.

Anthropometric and biochemical measurements are as follows: all subjects were evaluated with a series of anthropometric measurements and tests for hematology, biochemistry, and hormonal functions after an overnight fast. Biochemical measurements were performed at baseline for all 80 obese individuals and 20 LC and prospectively for the 40 MetS+ subjects in visits 2 and 3 of active weight loss.

All biochemical measurements were performed on frozen plasma samples obtained by centrifugation of freshly drawn blood (3000 × g for 20 minutes at 4°C) and subsequent storage at −70°C. Blood lipid profiles, including total cholesterol (TC, HDL cholesterol (HDL-C), calculated LDL cholesterol (LDL-C), TG), and nonesterified FA, as well as plasma *γ*-glutamyltransferase (GGT) concentrations, were determined by enzymatic assays using a Hitachi 911 autoanalyzer.

Lp-PLA_2_ activity was determined by colorimetric activity method using 1-myristoyl-2-(4-nitrophenylsuccinylphosphatidylcholine) as the enzyme substrate according to manufacturer's protocol (DiaDexus, Inc., San Francisco, CA, USA).

Plasma levels of ox-LDL were measured by a direct sandwich enzyme-linked immunoassay (Mercodia AB, Uppsala, Sweden), which uses the monoclonal capture antibody mAb-4E6 and a monoclonal detection antibody directed against a different epitope of the oxidized apoB molecule.

Plasma concentrations of MPO were determined by direct sandwich enzyme-linked immunoassay according to manufacturer's protocol (OXIS Research, Portland, OR, USA).

Plasma levels of insulin were determined on a Luminex-100 multianalyte profiling system using commercially available immunoassay panels (Linco Research, Inc., St. Charles, MO, USA). Measures of insulin resistance were obtained using the homeostasis model assessment of insulin resistance (HOMA-IR = fasting glucose (mg/dL) × fasting insulin (U/mL)/22.5) [45].

### 2.2. Statistical Analyses

Unless otherwise stated, data are expressed as mean ± standard error. Statistical analyses were performed using Stata version 11 (Stata, College Station, TX, USA). Differences in baseline characteristics between groups (MetS+, MetS−, LC) were tested using one-way analysis of variance (ANOVA) and post hoc tests for differences between groups, Fisher's exact test, or the Kruskal-Wallis test when the data were not normally distributed. MPO, glucose, insulin, HOMA-IR, and TG were log-transformed to account for their right-skewed distributions. Paired *t*-tests and Wilcoxon matched-pairs signed-rank tests were applied to assess changes in ox-LDL, MPO, Lp-PLA_2_ activity, and other metabolic factors among MetS+ subjects before and after weight loss. In order to maximize the data for analysis, any missing data points for certain metabolic factors were filled in using mean and regression imputation methods [44].

Multivariate linear regression and Pearson's correlation analysis were used to assess the independent effects of change in ox-LDL, MPO, Lp-PLA_2_ activity before and after the weight loss intervention. Percent changes were applied for the above parameters. The efficacy of the ox-LDL, MPO, and Lp-PLA_2_ activity tests in distinguishing groups of MetS+ versus MetS− was assessed by using logistic regression and receiver operating characteristic (ROC) analysis. *P* < 0.05 was considered to be statistically significant for all analyses.

## 3. Results

### 3.1. Baseline Characteristics

The baseline characteristics are described in Table 1. Average age of our study participants was 46 years (range, 30–71 years) at the time of recruitment. Sixty-eight percent of the participants were women. However, obese male participants were more likely to have MetS compared to obese female participants. The LC-group had normal weight and BMI, normal systolic blood pressure (SBP), diastolic blood pressure (DBP), normal glucose and insulin levels, and normal lipid profiles. Compared to the LC-group, MetS− and MetS+ patients had significantly higher SBP and DBP, higher levels of glucose, insulin, and HOMA-IR values. The MetS+ group had significantly higher levels of TG and lower levels of HDL when compared to the LC-group; MetS− subjects did not differ significantly from the LC-group in regard to their lipid profile.

**Figure 1:**
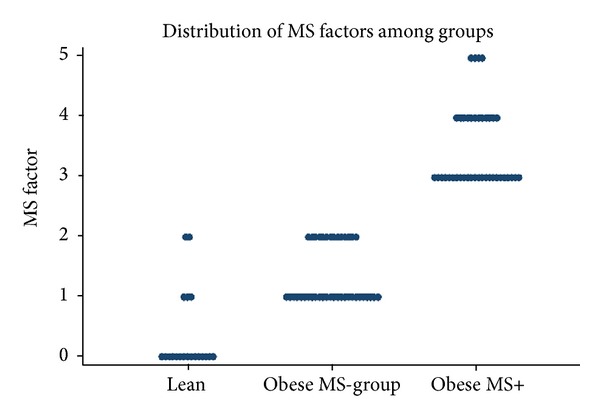
Distribution of metabolic syndrome factors among groups according to the NCEP ATPIII criteria.

**Figure 2:**
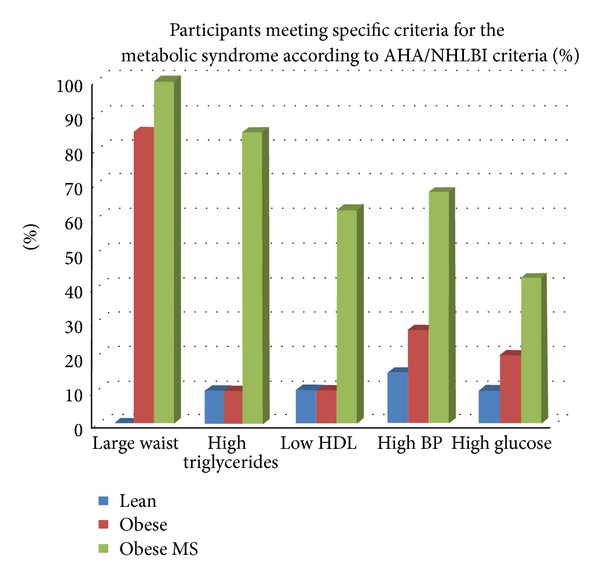
Prevalence of individual components of the metabolic syndrome among study groups based on the AHA/NHLBI criteria.

The distribution of MetS criteria among the participants based on the NCEP ATP III criteria is shown in Figure 1 and the percentage of participants in each group meeting the specific criteria for MetS based on the more recent AHA criteria is shown in Figure 2. In our study cohort, obese study participants were less likely to have elevated fasting blood glucose than any of the other MetS criteria.

### 3.2. Baseline Levels of Oxidative Stress Markers

At baseline MetS+ patients had significantly higher plasma ox-LDL levels when compared to the MetS-group (64.26 ± 2.2 versus 57.08 ± 1.5 U/L, *P* = 0.010) and LC-group (64.26 ± 2.2 versus 52.21 ± 3.0 U/L, *P* = 0.002), whereas circulating levels of MPO were significantly higher in obese patients (with or without MetS) when compared with the LC-group (330.79 ± 45.0 and 375.28 ± 65.1 versus 172.72 ± 22.0 pM, *P* = 0.015). Although Lp-PLA_2_ activity was the highest in the MetS+ group, we did not find significant differences between groups at baseline (Table 1). Baseline correlations (Pearson's regression) between oxidative stress markers and MetS components revealed that ox-LDL and Lp-PLA_2_ activity were adversely associated with cardiovascular lipid risk factors and showed a strong positive correlation with each other, while plasma MPO was associated with various measures of obesity and insulin resistance (Table 2).

**Figure 3:**
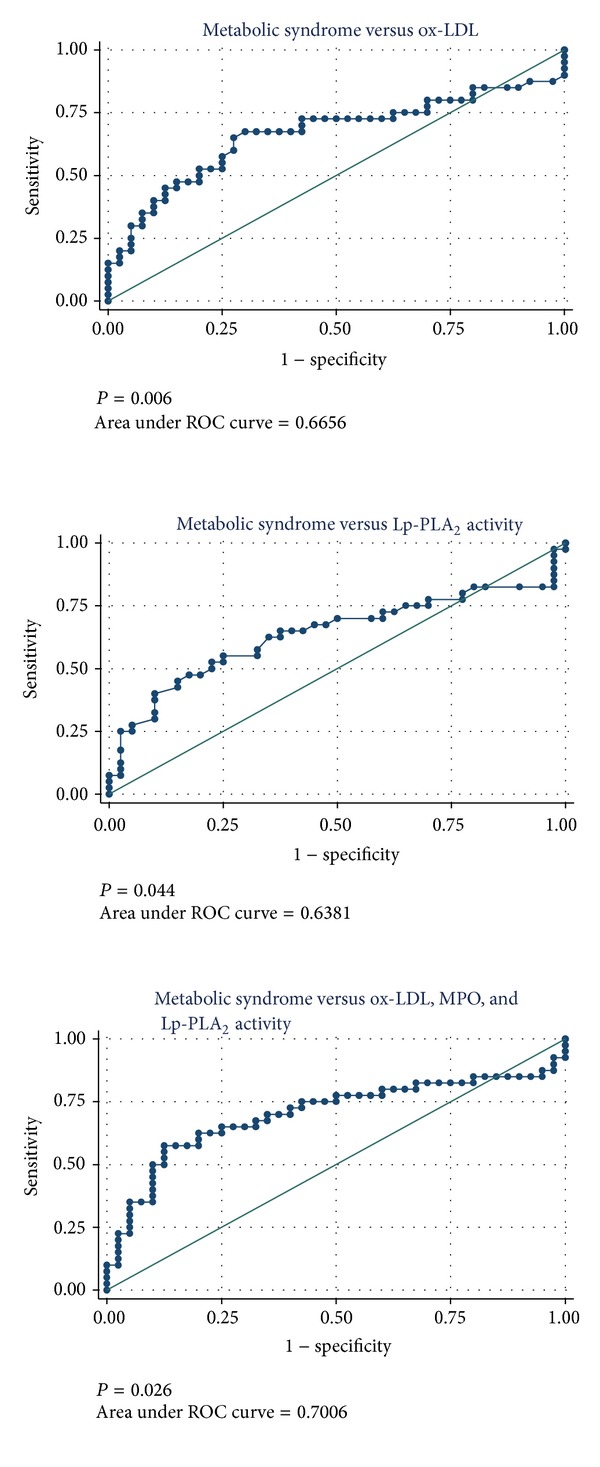
Relationship between oxidative stress markers and metabolic syndrome status in obese adults using ROC analysis.

We investigated the efficacy of the different oxidative stress markers to determine MetS status in obese individuals (Figure 3). Using logistic regression, we calculated that the odds ratio (OR) for MetS as predicted by ox-LDL was 1.06 (95% CI: 1.013–1.099, *P* = 0.010), indicating that for each 1 U/L increment in ox-LDL level the odds of having MetS increased by 6%. ROC analysis showed that the area under the ROC curve (AUC) for ox-LDL was 0.666. The OR for MetS as predicted by Lp-PLA_2_ activity was 1.02 (95% CI: 0.999–1.036, *P* = 0.051) and the AUC for Lp-PLA_2_ activity was 0.638. In contrast, MPO levels did not predict MetS status (OR = 1.00; 95% CI: 0.998–1.001, *P* = 0.548).

Multivariate logistic regression analysis showed that a model including ox-LDL, Lp-PLA_2_ activity, and MPO improved prediction of MetS status among obese individuals compared to each oxidative stress marker alone (AUC = 0.701, likelihood ratio test, *P* = 0.026). Although MPO did not predict MetS status alone, it did add incrementally to the AUC of a model including ox-LDL and Lp-PLA_2_ activity.

### 3.3. Effects of Weight Loss

**Figure 4:**
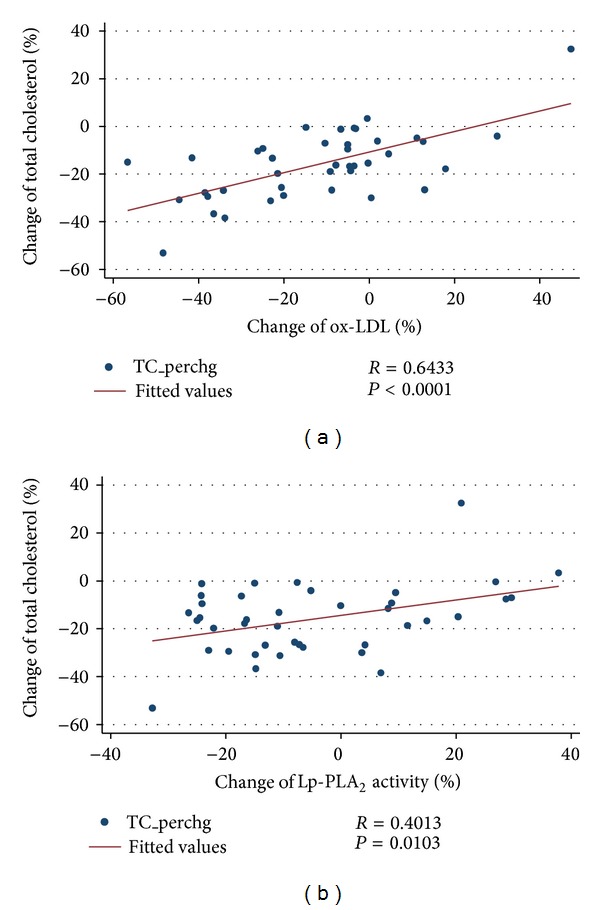
Percent change of total cholesterol versus percent change of ox-LDL and LpPLA_2_ activity following rapid weight loss among obese men and women with the metabolic syndrome, Pearson's correlation: (a) % change of total cholesterol versus % change of ox-LDL; (b) % change of total cholesterol versus % change of Lp-PLA_2_ activity.

**Figure 5:**
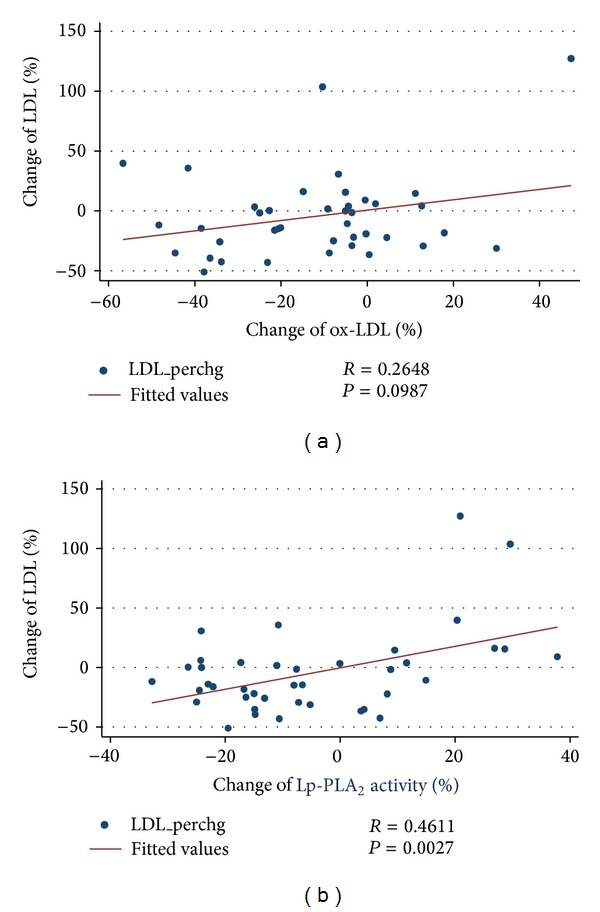
Percent change of LDL-C following rapid weight loss among obese men and women with the metabolic syndrome versus percent change of (a) ox-LDL and (b) Lp-PLA_2_ activity, Pearson's correlation.

The group of MetS+ subjects on the VLCD reached an average weight loss of 16.9 kg and a 5.6 kg/m^2^ decrease in BMI, which coincided with a 10% reduction in waist circumference (Table 3). Furthermore, MetS+ subjects showed a marked improvement in cardiometabolic risk factors, including blood pressure, lipid risk factors, fasting glucose, and insulin resistance following diet-induced weight loss. Rapid weight loss also led to changes in circulating levels of oxidative stress markers. VLCD resulted in a reduction of ox-LDL levels from 64.3 to 54.7 U/L. This 12% reduction (*P* < 0.001) was associated with a decrease in TC, even after adjustment for age and sex (*P* = 0.019). Dietary caloric restriction also led to a decrease in Lp-PLA_2_ activity by 4.7% from 136.6 to 127.7 nmol/mL/min (*P* = 0.024). The changes in ox-LDL and Lp-PLA_2_ activity associated with weight loss were positively correlated with changes in TC (Figures 4(a) and 4(b)) and LDL-C (Figures 5(a) and 5(b)). Rapid weight loss resulted in a 15% decrease in plasma MPO levels, although this reduction did not reach significance.

When we investigated the relationship between oxidative stress markers and glucose metabolism following rapid weight loss, we found a significant correlation of MPO with glucose (Pearson's *R* = −0.4317, *P* = 0.0054).

## 4. Discussion

In the current study, we confirmed findings from our previous report showing a significant improvement of metabolic factors following weight loss in subjects with MetS [42]. In addition, we found that baseline levels of individual oxidative stress markers did not consistently define MetS status. However, the combination of the three oxidative stress markers (i.e., ox-LDL, Lp-PLA_2_ activity, and MPO) significantly improved prediction of MetS status in our study cohort. Although Lp-PLA_2_ activity was the highest in the MetS+ group, we did not find significant differences between groups at baseline. Baseline ox-LDL levels were significantly higher in MetS+ obese individuals when compared to MetS− obese individuals and LC. This finding is in agreement with results from a previous study that reported elevated circulating ox-LDL levels in patients with MetS, whereas ox-LDL levels did not differ between obese MetS− individuals and LC [21]. We found that baseline MPO levels were significantly higher in obese individuals when compared with LC irrespective of presence of MetS, which suggests that MPO may reflect an increased oxidative stress linked to obesity. Taken together these findings indicate that an elevated oxidative stress status is present in obese individuals and particularly in those individuals with MetS.

There is a paucity of data from clinical studies investigating the role of oxidative stress in obesity and the effects of weight loss on oxidative stress status in metabolically healthy and metabolically abnormal individuals. Tzotzas et al. [46] found a significant reduction of Lp-PLA_2_ activity after low-calorie diet associated weight loss in healthy obese women. On the other hand, results of Hanusch-Enserer et al. [47], who examined the effect of weight loss after gastric banding surgery on cardiovascular risk factors, reported the Lp-PLA_2_ levels remained unchanged. Since diet-induced weight loss also resulted in a significant reduction of Lp-PLA_2_ activity in our study, the combined data from these studies suggest that different modes of therapeutic intervention to achieve weight loss may lead to differing effects on oxidative stress status.

Pierce et al. [48] investigated the influence of energy intake-restricted weight loss on endothelium-dependent artery dilation in overweight or obese nondiabetic men and women. This intervention resulted in a reduction of circulating ox-LDL levels. Shin et al. [49] also reported a decrease in circulating ox-LDL levels following diet-induced weight loss in metabolically abnormal obese individuals, although they found no reduction of ox-LDL levels in a group of healthy obese subjects. The fact that weight loss resulted in a significant reduction of circulating ox-LDL levels in obese MetS+ subjects in our study was in general agreement with the aforementioned studies.

Studying the effects of a 21-day diet and exercise intervention in men with MetS, Roberts and coworkers [50] noted significant improvements in lipid risk factors and HOMA-IR as well as a reduction in circulating MPO levels. Although differences in study population, dietary intervention, and time of follow-up exist between this study and ours, the general findings from both studies related to the effect of weight loss on metabolic parameters of lipid, glucose, and oxidative stress are similar in nature.

In summary, our results demonstrate that oxidative stress markers are predictive of MetS in obese individuals. Weight loss induced by dietary caloric restriction reduces oxidative stress concurrent with an improvement in the clinical components of MetS in these individuals. Obese individuals with MetS are in a state of hyperglycemia and chronic low-grade inflammation, conditions that promote increased production of ROS resulting in increased oxidative stress. Our data suggest that VLCD induced weight loss results in an improvement in glucose metabolism and may lead to decreased inflammation as a result of decreased adiposity and adipokine/cytokine secretion, thereby reducing the oxidative stress in these individuals. A limitation of our study is that we did not directly measure antioxidant capacity in study participants prior to or during the study. Although it is unlikely that study participants would experience decreased antioxidant capacity during the VLCD intervention given more than adequate dietary intake of antioxidant vitamins (140% DV), we can not rule out that the 140% DV of antioxidant vitamins could have enhanced the antioxidant capacity in individuals who may have had an insufficient antioxidant capacity thereby contributing to decreased levels of oxidative stress markers. Dietary caloric restriction resulted in a significant decrease in surrogate measures of body fat (i.e., BMI and waist circumference) in obese individuals. However, we did not investigate the effects of energy restriction on oxidative stress independent of changes in body fat mass in lean individuals. Future studies are needed to address the utility of oxidative stress markers as not only predictors of MetS in obesity but as markers of effective treatments that target cardiometabolic risk in the obese population.

## Supplementary Material

Criteria of the metabolic syndrome - American Heart Association/National Heart, Lung and Blood Institute (AHA/NHLBI) Scientific Statement.

## Figures and Tables

**Table 1:** Baseline characteristics among 20 lean controls and 80 obese adults with and without the metabolic syndrome (values are expressed as ^a^median (IQR), all others as means ± SE).

Characteristics	Lean	Obese		*P* value (3 groups)	*P* value (pairwise)		
	Controls (*n* = 20)	MS− (*n* = 40)	MS+ (*n* = 40)		LC versus MS−	LC versus MS+	MS− versus MS+
Age (years)	44.35 ± 2.0	46.85 ± 1.0	48.05 ± 1.6	0.2823^b^	0.280^c^	0.113^c^	0.527^c^
Gender (% female)	60	83	58	0.0388^b^	0.076^c^	0.842^c^	0.016^c^
Weight (kg)	66.80 ± 2.6	103.65 ± 3.3	116.84 ± 3.8	<0.0001^b^	<0.001^c^	<0.001^c^	0.006^c^
BMI (kg/m^2^)	22.98 ± 0.6	37.85 ± 0.9	38.81 ± 1.0	<0.0001^b^	<0.001^c^	<0.001^c^	0.444^c^
Waist (in)	30.43 ± 0.6	41.39 ± 0.9	45.32 ± 1.1	<0.0001^b^	<0.001^c^	<0.001^c^	0.003^c^
SBP (mmHg)	112.95 ± 2.8	120.3 ± 2.0	136.63 ± 2.9	<0.0001^b^	0.080^c^	<0.001^c^	<0.001^c^
DBP (mmHg)	68.45 ± 2.0	78.9 ± 1.4	84.8 ± 1.5	<0.0001^b^	<0.001^c^	<0.001^c^	0.005^c^
Glucose^a^ (mg/dL)	87.0 (81.5, 93.0)	94.0 (87.0, 99.0)	97.5 (88.5, 107.5)	0.0025	0.032	0.001	0.058
Insulin^a^ (mU/L)	4.53 (3.63, 7.42)	12.57 (6.85, 16.91)	17.44 (11.04, 27.69)	0.0001	<0.001	<0.001	0.004
HOMA-IR^a^	0.95 (0.82, 1.58)	2.66 (1.62, 4.14)	4.23 (2.63, 11.29)	0.0001	<0.001	<0.001	0.002
Triglycerides^a^ (mg/dL)	90.5 (66.5, 127.5)	110.5 (91.5, 133.0)	225.5 (176.0, 292.5)	0.0001	0.086	<0.001	<0.001
HDL (mg/dL)	60.43 ± 3.1	61.15 ± 2.7	44.1 ± 1.7	<0.0001^b^	0.853^c^	<0.001^c^	<0.001^c^
LDL (mg/dL)	119.21 ± 7.2	125.38 ± 4.2	121.91 ± 5.5	0.7409^b^	0.459^c^	0.749^c^	0.620^c^
Total cholesterol (mg/dL)	201.5 ± 8.7	205.98 ± 5.3	211.15 ± 6.1	0.6120^b^	0.658^c^	0.340^c^	0.530^c^
ox-LDL (U/L)	52.21 ± 3.0	57.08 ± 1.5	64.26 ± 2.2	0.0011^b^	0.153^c^	<0.001^c^	0.011^c^
MPO^a^ (pM)	163.1 (84.4, 259.7)	250.1 (181.7, 392.8)	240.3 (164.3, 393.2)	0.0253	0.015	0.013	0.927
Lp-PLA_2_ activity (nmol/mL/min)	128.09 ± 9.3	125.57 ± 3.4	136.58 ± 4.9	0.2600^b^	0.764^c^	0.307^c^	0.113^c^
MS factor^a^	0.00 (0.00, 1.00)	1.00 (1.00, 2.00)	3.00 (3.00, 4.00)	0.0001	<0.001	<0.001	<0.001

*P* values calculated using ^a^the Kruskal-Wallis test, ^b^one-way ANOVA, and ^c^post-ANOVA pairwise comparison of means.

**Table 2:** Baseline correlations (Pearson's regression) of several parameters with ox-LDL, MPO, and Lp-PLA_2_ activity in subjects with and without the metabolic syndrome (*n* = 100).

Variable	ox-LDL (U/L)		MPO^a^ (pM)		Lp-PLA_2_ activity (nmol/mL/min)	
	*R*	*P*	*R*	*P*	*R*	*P*
Weight (kg)	0.195	0.056	0.275	0.007	0.203	0.047
Waist (in)	0.202	0.052	0.253	0.015	0.234	0.024
BMI (kg/m^2^)	0.128	0.212	0.300	0.003	0.062	0.544
SBP (mmHg)	0.092	0.372	0.078	0.445	0.015	0.883
DBP (mmHg)	0.196	0.055	0.023	0.822	0.066	0.523
Glucose^a^ (mg/dL)	−0.033	0.749	0.095	0.357	0.106	0.303
Insulin^a^ (mU/L)	0.153	0.134	0.296	0.003	0.064	0.536
HOMA-IR^a^	0.129	0.207	0.283	0.005	0.079	0.445
Triglycerides^a^ (mg/dL)	0.479	0.000	−0.018	0.858	0.292	0.004
HDL (mg/dL)	−0.231	0.023	−0.108	0.292	−0.404	0.000
LDL (mg/dL)	0.629	0.000	−0.001	0.993	0.309	0.003
Total cholesterol (mg/dL)	0.687	0.000	−0.052	0.612	0.297	0.003
ox-LDL (U/L)	—	—	0.004	0.968	0.378	0.000
MPO^a^ (pM)	0.004	0.968	—	—	0.117	0.254
Lp-PLA_2_ Activity (nmol/mL/min)	0.378	0.000	0.117	0.254	—	—

^a^Variables are not normally distributed. Log-transformed value before regression.

**Table 3:** Change in metabolic factors^b^ following rapid weight loss among 40 obese adults with the metabolic syndrome (values are expressed as means ± SE).

Metabolic factors	Baseline	Final	Change	Percent change	*P*
Weight (kg)	116.84 ± 3.8	99.92 ± 3.2	−16.92 ± 1.1	−14.32 ± 0.7	<0.001
Waist (in)	45.14 ± 1.0	40.53 ± 1.1	−4.61 ± 0.5	−10.23 ± 1.0	<0.001
BMI (kg/m^2^)	38.81 ± 1.0	33.17 ± 0.8	−5.64 ± 0.3	−14.32 ± 0.7	<0.001
SBP (mmHg)	136.63 ± 2.9	128.04 ± 2.0	−8.58 ± 2.0	−5.50 ± 1.4	<0.001
DBP (mmHg)	84.8 ± 1.5	79.39 ± 0.7	−5.41 ± 1.4	−5.33 ± 1.7	0.001
Glucose^a^ (mg/dL)	108.95 ± 6.7	90.77 ± 2.1	−18.18 ± 7.2	−10.04 ± 3.3	0.004
Insulin^a^ (mU/L)	27.40 ± 4.1	11.30 ± 2.2	−16.09 ± 4.4	−36.77 ± 14.8	<0.001
HOMA-IR^a^	7.54 ± 1.2	2.53 ± 0.5	−5.01 ± 1.2	−43.02 ± 13.5	<0.001
Triglycerides^a^ (mg/dL)	254.7 ± 24.4	123.76 ± 8.2	−130.94 ± 24.7	−38.09 ± 6.1	<0.001
HDL (mg/dL)	44.1 ± 1.7	38.57 ± 1.5	−5.53 ± 1.1	−11.11 ± 2.6	<0.001
LDL (mg/dL)	121.18 ± 5.2	109.20 ± 4.6	−11.97 ± 5.7	−4.51 ± 5.6	0.041
Total cholesterol^a^ (mg/dL)	211.5 ± 6.1	174.87 ± 5.3	−36.28 ± 5.3	−16.00 ± 2.3	<0.001
ox-LDL (U/L)	64.26 ± 2.2	54.69 ± 1.8	−9.57 ± 2.4	−11.97 ± 3.4	<0.001
MPO^a^ (pM)	330.79 ± 45.0	275.85 ± 34.9	−54.93 ± 50.5	−15.36 ± 14.8	0.354
Lp-PLA_2_ activity (nmol/mL/min)	136.58 ± 4.9	127.65 ± 4.4	−8.93 ± 3.8	−4.72 ± 2.9	0.024

^a^ *P* value calculated using Wilcoxon matched-pairs signed-rank tests; all others using paired *t*-tests.

^b^Based on data from visit 3. Missing data was filled in by applying the multiple imputation methods.
