# Supplementary material for: The Impact of Rapid Weight Loss on Oxidative Stress Markers and the Expression of the Metabolic Syndrome in Obese Individuals
Source: J Obes. 2013 Dec 19;2013:729515. doi: 10.1155/2013/729515 (PMC3880717; doi:10.1155/2013/729515)
Supplement: Supplementary file 1 — Criteria of the metabolic syndrome - American Heart Association/National Heart, Lung and Blood Institute (AHA/NHLBI) Scientific Statement. [file 729515.f1.pdf]

Supplemental Table 1: Criteria of the metabolic syndrome - American Heart Association/National Heart, Lung and Blood Institute (AHA/NHLBI) Scientific Statement

**Risk factor**	**Defining level**
Waist circumference	
Men	>40 in (>102 cm)
Women	>35 in (>88 cm)
Triglycerides	≥150 mg/dl
HDL cholesterol	
Men	<40 mg/dl
Women	<50 mg/dl
Blood pressure	≥130/≥85 mm Hg
Fasting glucose	≥100 mg/dl
